# Need for homeostasis in electrical activity may account for cortical network rewiring

**DOI:** 10.1186/1471-2202-12-S1-F1

**Published:** 2011-07-18

**Authors:** Markus Butz, Arjen van Ooyen

**Affiliations:** 1 Department of Integrative Neurophysiology, Computational Neuroscience Group, VU University Amsterdam

## 

Current thinking about how the brain learns from experience and encodes memories is focused on changes in the strengths of connections between neurons, whereby the pattern of connections is considered fixed. However, the adult brain is not as hard-wired as traditionally thought. Neurons establish new connections and break existing ones (structural plasticity). Although structural plasticity associated with brain lesions (stroke) and degeneration is known since the late 1960s, its relevance for cognitive functions such as learning and memory [4,8] and its contribution to network repair after lesions [5,7,9] still remain unclear. Current computational models are insufficient to address this issue since network structure is considered as fixed with plasticity merely arising from changes in connection strengths of existing synapses. The present work aims to elucidate the underlying organizing principles that drive structural plasticity and brain repair induced by lesions. We hypothesize that the need of neurons to maintain their average electrical activity at a particular level (homeostatic regulation) drives lesion-induced restructuring of cortical circuits, and can predict changes in connectivity and spine/bouton/synapse numbers experimentally observed following cortical deafferentations such as a focal retinal lesion [5,9]. In this view, loss of input due to lesions disturbs the homeostasis of neuronal activity, which, through activity-dependent neurite outgrowth and synapse formation, triggers compensatory structural changes that attempt to regain homeostasis [2].

To explore whether the experimentally observed structural changes can be accounted for by the neurons’ need for homeostasis in electrical activity, we constructed a neuronal network model in which each neuron receives a vertical input stream (from the eye via the thalamus) and a horizontal input stream (from other neurons within the cortical network). The focal retinal lesion is modeled as a circumscribed removal of the vertical input stream. To model structural changes of the neuron, we created a novel model formalism in which each neuron has a number of axonal elements (representing boutons) and dendritic elements (representing spines) [1,3]. Synapses are formed by merging axonal and dendritic elements. In line with the experimental data, the number of these elements can change depending on the neuron’s own level of electrical activity, which may cause existing synaptic connections to break or new ones to form. A neuron will change the number of its elements so as to try to maintain its average activity at a particular set-point. So, when its activity is above the set-point, it will eliminate (dendritic) elements, and when activity is below the set-point it will generate new elements. However, when activity is too low, neurons will also lose axonal and dendritic elements. Electrical activity is generated by a conductance-based spiking neuron model with intracellular calcium as a measure of the average electrical activity of the single neuron [6].

By this approach we could show that the neuronal need for homeostasis in electrical activity can account for the structural changes observed in the visual cortex after focal retinal lesions [5,9]. We hypothesize that the precise interplay between axons and dendrites as well as the topology of the network matter for the experimental results. In this study, we investigated different scenarios depending on the calcium-dependency of axonal growth, which may give insight into potential approaches for promoting network repair in experimental deafferentation studies. In addition to making testable predictions, our network model is the first to describe the reciprocal dynamics of activity-dependent network rewiring. Our findings on topological changes are in line with recent data from stroke patients [7] and may imply a more general principle of the mechanisms involved in brain repair. A better understanding of neuronal network repair following lesions is urgently needed for a better treatment of neurological diseases such as stroke.
